# Risk of perinatal death and preterm birth among an observational cohort of women vaccinated against SARS-CoV-2 in pregnancy: CDC COVID-19 vaccine pregnancy registry

**DOI:** 10.1016/j.vaccine.2026.128461

**Published:** 2026-03-14

**Authors:** Sabrina A. Madni, Christine K. Olson, Lauren Head Zauche, Aliza Machefsky, Ansley V. Waters, Reji Padathara-Mathew, Victoria Okereke, Andrea J. Sharma

**Affiliations:** a Division of Healthcare Quality Promotion, National Center for Emerging and Zoonotic Infectious Diseases, CDC, Atlanta, GA, USA; b U.S. Public Health Service Commissioned Corps, Rockville, MD, USA; c Department of Gynecology and Obstetrics, Emory University School of Medicine, Atlanta, GA, USA; d Deloitte Consulting LLP, Arlington, VA, USA; e Eagle Global Scientific, LLC, Atlanta, GA, USA

**Keywords:** COVID-19, Pregnancy, Vaccination, Pregnancy outcome, Preterm birth, Perinatal death

## Abstract

**Background:**

COVID-19 vaccine clinical trials excluded pregnant women; additional data on safety of COVID-19 vaccines in pregnancy can strengthen clinical recommendations.

**Objective:**

To assess risk of perinatal death (stillbirth and neonatal death), and preterm birth among women receiving COVID-19 vaccines just prior to or during pregnancy.

**Methods:**

The Centers for Disease Control and Prevention's COVID-19 Vaccine Pregnancy Registry (C19VPR) is an observational cohort of women receiving ≥1 COVID-19 vaccine between December 15, 2020, and June 21, 2021, up to 30 days before their last menstrual period or during pregnancy. Data were collected through interviewer-administered phone surveys. We matched C19VPR pregnancies to 2019 US National Vital Statistics System (NVSS) data based on race, ethnicity, and age group. We estimated week-specific and cumulative risk of perinatal death and preterm birth using a fetuses-at-risk approach among singleton pregnancies ending ≥6 weeks' gestation. For C19VPR participants, pregnancies entered the risk pool at gestational age of vaccination. We compared C19VPR estimates to NVSS using Cox regression to calculate hazard ratios and 95% confidence intervals adjusted for race, ethnicity, age, and pre-pregnancy body mass index. Among C19VPR pregnancies, we used Cox regression to assess difference in risk by vaccine manufacturer.

**Results:**

Cumulative risk of perinatal death among C19VPR pregnancies and NVSS were 4.8 and 8.1 per 1000 births, respectively. Cumulative risk of preterm births among C19VPR pregnancies and NVSS were 6.6% and 12.1%, respectively. Adjusted hazard ratios (aHR) suggested lower cumulative risk of perinatal death and preterm birth among C19VPR pregnancies (vaccinated) compared with 2019 NVSS (pre-pandemic estimate) (aHR 0.62 95%CI 0.46, 0.83; aHR 0.55 95%CI 0.51, 0.58, respectively). Within the C19VPR cohort, risks of perinatal death and preterm birth did not differ by vaccine manufacturer.

**Conclusion:**

We found no evidence of increased risk of perinatal death or preterm birth after COVID-19 vaccination in pregnancy.

## 1. Introduction

Data from early in the COVID-19 pandemic suggested that pregnant women are more likely to experience severe COVID-19 illness compared with non-pregnant women [1]. Some studies identified an increased risk of adverse pregnancy outcomes, including preterm birth, stillbirth, and neonatal death, among pregnant women infected with severe acute respiratory syndrome coronavirus 2 (SARS-CoV-2) [2–7]. Receipt of mRNA COVID-19 vaccine products has been associated with a lower risk of COVID-19 associated severe illness and hospitalization [8,9]. COVID-19 vaccination during pregnancy reduces the risk of severe illness and complications associated with SARS-CoV-2 infection during pregnancy [10–12]. The American College of Obstetricians and Gynecologists (ACOG) recommended that eligible pregnant women receive COVID-19 vaccines in pregnancy [13]. Pregnant women in the US remain less likely to receive the COVID-19 vaccine than other vaccines routinely recommended in pregnancy [14]. Reports have indicated that COVID-19 vaccine hesitancy among pregnant women is due in part to limited data on COVID-19 vaccine safety in pregnancy both in the United States and internationally [15–18]. Existing studies examining associations between COVID-19 vaccination in pregnancy and adverse perinatal outcomes have found no increased risk of adverse pregnancy outcomes [10,11,19–29]. However, some studies are limited by small sample sizes [21–25], and few participants who were vaccinated early in pregnancy [11,21,26–28]. Additional data about associations of COVID-19 vaccines with adverse perinatal outcomes can strengthen the evidence pool and inform decisions and recommendations about COVID-19 vaccination in pregnancy among pregnant women and healthcare providers, respectively. Much of the existing evidence comes from international settings. Additional US-based data can inform US vaccine policy, as healthcare infrastructure and access, which are associated with perinatal death and preterm birth, differ between countries.

In January 2021, the CDC established the COVID-19 Vaccine Pregnancy Registry (C19VPR) as part of comprehensive vaccine safety monitoring [30]. The registry collected data on COVID-19 vaccination in pregnancy, participant health, pregnancy outcomes, and infant health among women who received at least one COVID-19 vaccine up to 30 days prior to last menstrual period or during pregnancy. To understand the risk of adverse perinatal outcomes after COVID-19 vaccination, this analysis compares risks of preterm birth, stillbirth, and neonatal death among C19VPR pregnancies to 2019 National Vital Statistics System (NVSS) data.

## 2. Methods

### 2.1. Study design and cohort selection

To collect participant-reported symptoms and health impacts following COVID-19 vaccination during the COVID-19 pandemic, CDC launched V-safe, an active vaccine safety monitoring system, in December 2020 [31]. V-safe asked women whether they were pregnant after each reported vaccine. V-safe participants reporting a pregnancy between December 15, 2020, and June 21, 2021, were screened for C19VPR eligibility. C19VPR staff reviewed vaccination information reported to V-safe with potential participants and collected information on pregnancy timing relative to vaccination. Eligible pregnancies included those reported by women aged 18–54 years with vaccination up to 30 days prior to the last menstrual period (LMP) or during pregnancy. Detailed information about C19VPR methodology is available [30]. C19VPR participants were interviewed up to 5 times during and after pregnancy from January 2021 through September 2023. This activity was reviewed by CDC, deemed not research, and was conducted consistent with applicable federal law and CDC policy, 45C.F.R. part 46.102(l)(2), 21C.F.R. part 56; 42 U.S.C. §241(d); 5 U.S.C. §552a; 44 U. S.C. §3501 et seq..

### 2.2. Exclusion criteria

There were 23,265 eligible pregnancies among the 23,249 participants enrolled in C19VPR; sixteen participants (0.7%) reported two registry-eligible pregnancies [30]. Consistent with previously published literature, we excluded multiple gestation pregnancies (*n* = 426), pregnancy outcomes reported to have occurred prior to 6 weeks gestation (*n* = 154), and pregnancies lost to follow-up prior to 6 weeks gestation (*n* = 3) [32–34]. The final sample included 22,682 pregnancies.

### 2.3. Comparison group

The C19VPR cohort includes only vaccinated participants; thus, we matched C19VPR pregnancies to 2019 NVSS data (i.e., 2019 fetal death data and 2019–2020 linked infant birth and death data) by participant age groups and condensed race and ethnicity groups using coarsened exact matching [35,36].

### 2.4. Exposure

The exposure of interest was COVID-19 vaccination in the 30 days prior to LMP preceding pregnancy or during pregnancy. At the time of C19VPR participant identification, only the first monovalent (original) virus COVID-19 vaccines formulated for the original SARS-CoV-2 variant were available (i.e., Pfizer-BioNTech's BNT162b2, Moderna's mRNA-1273, and Janssen's Ad26.COV2. S). For 96.7% of participants, the registry-eligible dose was also the first COVID-19 vaccine dose received, given the timing of C19VPR enrollment relative to vaccine availability.

### 2.5. Outcomes

Outcomes analyzed included perinatal death and preterm birth. We asked participants how their pregnancy ended; specific choices included (1) “miscarriage,” (2) “stillbirth,” (3) “induced abortion,” (4) “live birth,” or (5) “another outcome including ectopic or molar pregnancy.” We also asked all participants to provide the date and gestational age at which their pregnancy ended. For participants reporting a live birth, we asked whether their infant was still living and, if applicable, date of infant death. C19VPR survey questions utilized in this analysis can be found in Supporting Information Supplement 1.

To ascertain the most precise pregnancy dating possible, we calculated analytic gestational age at the end of pregnancy in weeks (([end of pregnancy date]—[LMP])/7). If dates were not reported, participant-reported gestational age at the end of pregnancy was used. To mitigate competing risks, we combined stillbirths and neonatal deaths into perinatal deaths [37]. CDC's National Vital Statistics System (NVSS) has historically used two definitions of perinatal death, one including neonatal deaths up to 7 days after birth and one including neonatal deaths up to 28 days after birth [38]. We used the 28-day definition to maximize sensitivity for adverse outcomes. Stillbirth was defined as any intrauterine fetal demise or delivery of a fetus with a known or presumed gestational age of 20 weeks or greater and no signs of life (e.g., heartbeat, respirations, voluntary muscle movements, and pulsations of umbilical cord) [39]. Live birth was defined as the complete expulsion or extraction of a product of human conception, regardless of duration of pregnancy, wherein signs of life are present [40]. Preterm birth was defined as live birth prior to 37 completed weeks of gestation. Infants could be classified as both a preterm live birth and neonatal death.

### 2.6. Confirmation of pregnancy outcomes

Previous research has demonstrated discrepancies between participant report of stillbirth and pregnancy outcome as documented in medical records (e.g., reporting a spontaneous abortion between 17 and 19 weeks 6 days' gestation as a stillbirth) [41]. To confirm participant report of pregnancy outcome, we requested consent for medical record release to study investigators [30]. The majority (84.4%) of participants consented to medical record release [30]. To note, medical record consent was less common among women who were nulliparous, ≥40 years of age, non-Hispanic Black or not reporting race and ethnicity, and women who reported pregnancy outcome of miscarriage.

SABs ≥17 weeks' gestation, stillbirths, and neonatal deaths occurring within 1 day of delivery were initially characterized as “possible stillbirths.” Among consenting participants, two clinicians reviewed medical records independently to identify the pregnancy outcome. Reviews were compared, and the clinical determination was discussed in the event of disagreement until a consensus was met. If consent was provided but medical records were not received, C19VPR clinicians called clinics to confirm pregnancy outcomes. After review, pregnancy outcomes were reclassified if necessary.

### 2.7. Statistical analysis

Descriptive statistics were calculated to evaluate whether preterm births and perinatal deaths in the C19VPR cohort displayed similar demographic trends to the general US population. We evaluated covariates for potential confounding that may bias the observed association between vaccination and adverse pregnancy outcomes. Covariate data were reported by participants during standardized interviews conducted by C19VPR staff. Covariates included participant age at vaccination, race and ethnicity, pre-pregnancy body mass index (BMI), urban residence, chronic hypertension diagnosed prior to pregnancy, parity, timing of first vaccination relative to pregnancy (i.e., pre-pregnancy, first, second, or third trimesters) and history of the adverse outcome under examination (e.g., preterm birth).

For all outcomes, we assessed for effect modification by vaccine manufacturer. Cox proportional hazards regression was used to calculate hazard ratios adjusted for variables associated with both adverse outcomes and vaccine manufacturer. Participants receiving Janssen were excluded from manufacturer-specific analyses due to the small proportion receiving the product. Participants receiving doses 1 and 2 from different manufacturers were excluded from manufacturer-specific analyses (*n* = 10).

We used a fetuses-at-risk approach to estimate week-specific and cumulative risks of perinatal death and preterm birth for both C19VPR and the matched NVSS cohort [33,37]. For C19VPR pregnancies, left censoring was used to ensure fetuses did not enter the analysis until the gestational week at which the registry-eligible vaccine dose was received. For both C19VPR and NVSS pregnancies, censoring was used to ensure that fetuses did not contribute to the analysis after a pregnancy outcome had occurred. Other pregnancy outcomes (e.g., spontaneous abortion) were censored from the analysis at gestational age of pregnancy end [42]. Some C19VPR pregnancies had no specified date or gestational age of outcome or were lost to follow-up (i.e., pregnancy outcome unknown); these pregnancies were censored from the analysis at gestational age of last contact. Perinatal death analyses included all 22,682 C19VPR pregnancies and matched NVSS pregnancies. C19VPR pregnancies wherein all COVID-19 vaccines were received after 37 weeks' gestation were excluded from preterm birth analyses (*n* = 479), resulting in a subsample of 22,203 C19VPR pregnancies. Based on the 2019 national prevalence of 9.3 perinatal deaths per 1000 live births and stillbirths [35], we had 81% power (alpha = 0.05) to detect a 20% difference in perinatal deaths, our rarest outcome.

To compare C19VPR (vaccinated) risk estimates to 2019 NVSS (pre-pandemic estimate), we used Cox regression to calculate hazard ratios and 95% confidence intervals, adjusting for further stratified race and ethnicity groups (non-Hispanic [NH] Black, NH White, Hispanic, NH American Indian or Alaskan Native, NH Native Hawaiian or Pacific Islander, NH Asian, NH multi-racial, or unknown), age (continuous), and pre-pregnancy BMI (above or below 30). Matching variables were included in the model because coarsened matching was used; inclusion of matching variables in the model controls for residual confounding present due to group-based matching [43]. Perinatal epidemiologists have documented reasons for both combining and independently assessing risk of stillbirth and neonatal death [37,44]. Combining mitigates competing risk of outcomes, while independently assessing risk may be more appropriate due to different outcome etiologies [37,44]. Thus, we also provided independent cumulative risk estimates and Cox proportional hazards for stillbirth and neonatal death. C19VPR pregnancies entered the model at gestational week of vaccination. All NVSS pregnancies entered the model immediately as NVSS pregnancies did not receive the exposure of interest (COVID-19 vaccine) and, thus, did not require left censorship for entry into the model after exposure. We calculated *E*-values for the point estimate and for the limit of the confidence interval closest to the null to assess the potential impact of unmeasured confounding on observed associations, using the published formula: E-value=RR+√[RR×(RR–1)] for risk ratios greater than 1, and the corresponding transformation for risk ratios less than 1 [45]. All analyses were conducted using SAS (version 9.4, SAS Institute).

## 3. Results

Among the 22,682 C19VPR pregnancies included in this analysis, most participants were non-Hispanic White (79.1%), aged 30–34 years (48.4%), and reported urban residency (93.5%) (Table 1). The majority reported vaccine manufacturer as Pfizer-BioNTech (58.0%), followed by Moderna (38.9%) and Janssen (3.1%) (Table 1). Among participants who received mRNA vaccines, 10 participants received doses 1 and 2 from different manufacturers. Compared to participants receiving Pfizer-BioNTech vaccines, more participants receiving Moderna vaccines were Hispanic or non-Hispanic Black, reported rural residence, had BMI ≥30, and were vaccinated prior to 4 weeks' gestation (data not shown). In total, 95 pregnancy outcomes were categorized as “possible stillbirths” and reviewed by C19VPR clinicians; eleven (11.6%) were reclassified (Supplement 2).

### 3.1. Risk of perinatal death

**Fig. 1.**
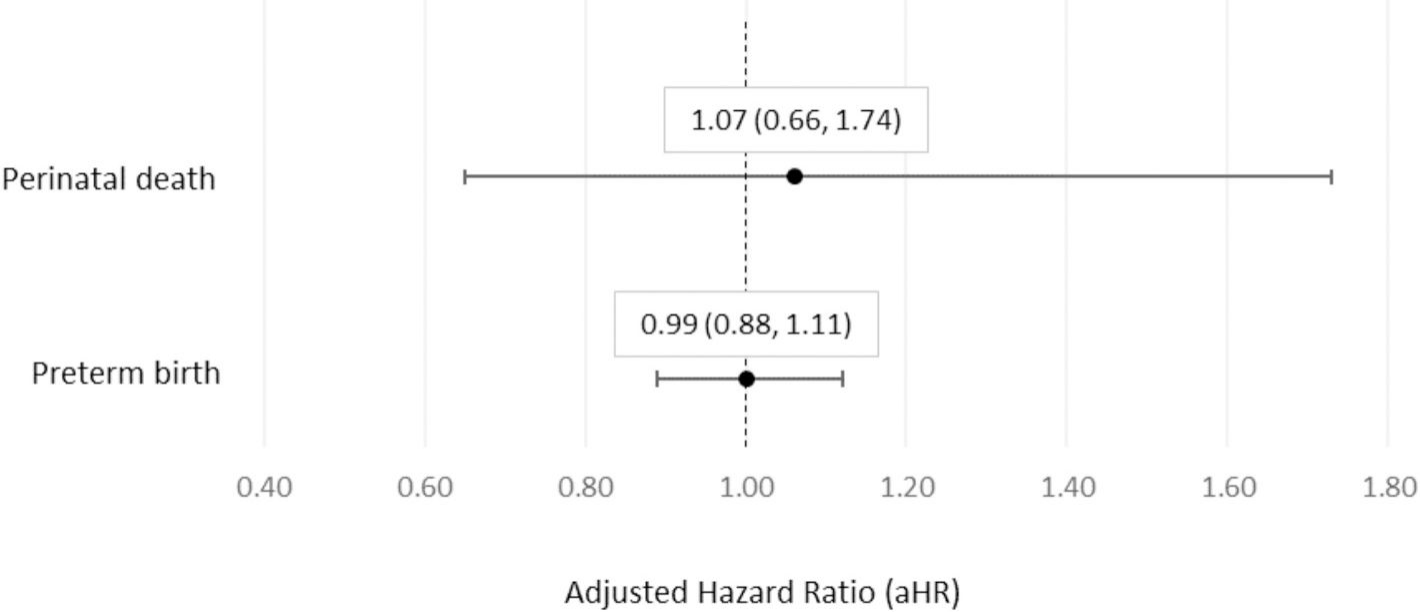
Adjusted hazard ratios of perinatal death and preterm birth comparing Moderna (mRNA-1273) to Pfizer-BioNTech (BNT162b2) among pregnancies in the CDC COVID-19 Vaccine Pregnancy Registry,^a^ January 2021 – August 2022. ^a^Hazard ratios are adjusted for maternal race and ethnicity, age at vaccination, timing of vaccination in pregnancy, pre-pregnancy BMI, urban residence, pre-existing high blood pressure, parity, and previous incident of outcome of interest (i.e., previous preterm birth for the preterm birth analysis). Pfizer-BioNTech is the reference group.

Cumulative risk of perinatal death was 4.79 (95% CI 3.57, 6.01) per 1,000 live births and stillbirths from 18 through 42 weeks' gestation among C19VPR pregnancies (Table 2). The earliest reported perinatal death was a live birth occurring at 18 weeks' gestation followed by neonatal death (also included in the preterm birth model). We found no significant difference in risk of perinatal death among participants receiving Moderna COVID-19 vaccines compared to those receiving Pfizer-BioNTech (adjusted HR 1.07; 95% CI 0.66, 1.74) (Fig. 1). Week-specific risk of perinatal death in the C19VPR cohort was below or similar to 2019 NVSS data at all gestational age intervals (Table 2). Cumulative risk of perinatal death was 38% lower among C19VPR pregnancies compared with NVSS when adjusting for maternal pre-pregnancy BMI, race and ethnicity, and age (aHR 0.62; 95% CI 0.46, 0.83) (Table 3). We found an *E*-value of 2.55 for the point estimate and 1.70 for the limit of the confidence interval closest to the null. Further, when compared with NVSS, cumulative risks of stillbirth (aHR 0.81; 95% CI 0.57, 1.14) and neonatal death (aHR 0.33; 95% CI 0.18, 0.69) were each independently lower in the C19VPR cohort, though results were not significant for stillbirth (Table 3).

### 3.2. Risk of preterm birth

Among pregnancies wherein participants received at least one dose of COVID-19 vaccine prior to 37 weeks' gestation (*n* = 22,203), the cumulative risk of preterm birth was 6.57% (95% CI 6.23, 6.92) (Table 4). We found no significant difference in risk of preterm birth among participants receiving Moderna compared to those receiving Pfizer-BioNTech (aHR 0.99; 95% CI 0.88, 1.18) when adjusting for maternal age, race and ethnicity, urban residence, hypertensive disorders of pregnancy, and pre-pregnancy BMI (Fig. 1). Cumulative risk of preterm birth was 45% lower among C19VPR pregnancies compared with NVSS when adjusting for maternal pre-pregnancy BMI, race and ethnicity, and age (aHR 0.55; 95% CI 0.51, 0.58) (Table 3). We found an *E*-value of 3.04 for the point estimate and 2.84 for the limit of the confidence interval closest to the null. Compared to NVSS, week-specific risk of preterm birth was observed to be lower or the same in the C19VPR cohort at all gestational weeks (Table 4) [35].

## 4. Discussion

[45] In this study, we did not identify an increase in risk of perinatal death or preterm birth after COVID-19 vaccination in pregnancy compared with pre-pandemic estimates. Our findings are comparable to those from previous reports, which identified no increased risk of preterm birth, stillbirth, or neonatal death after COVID-19 vaccination in pregnancy [22–28,46] A matched cohort study from Scotland of 81,441 singleton pregnancies that reached at least 20 weeks' gestation found [46] A matched case-control analysis powered to assess up to 50% increased risk found no association between stillbirth and COVID-19 vaccination in pregnancy [25] An Israeli study found no association between Pfizer-BioNTech (BNT162b2) COVID-19 vaccination [28].

Findings in this report are specific to original COVID-19 vaccine formulations, as participants were vaccinated from December 2020 through June 2021. Few studies have assessed risk of adverse pregnancy outcomes following receipt of COVID-19 vaccines formulated after the original virus COVID-19 vaccines. However, compared to original virus mRNA COVID-19 vaccines, newer mRNA COVID-19 vaccines are formulated using similar processes [47]. A report comparing Moderna's mRNA-1273.214 vaccine (bivalent vaccine released in August 2022) to the mRNA-1273 vaccine (original virus formulation) among men and non-pregnant women identified similar safety profiles [48].

Our analysis has several strengths. First, compared to other US-based studies assessing risk of preterm birth or perinatal death [22,27], we had a larger sample of women vaccinated during pregnancy. Assessment of risk of perinatal death is challenging, as rare outcomes require large sample sizes to be useful. With over 20,000 pregnancies, we were able to examine risk of perinatal death, a rare outcome. Second, detailed data on timing of vaccination during pregnancy enabled us to calculate week-specific risk, with pregnancies entering the risk pool only after participants were vaccinated. Week-specific and cumulative risk based on the fetuses-at-risk approach are more precise measures than overall incidence, as the weekly denominator is adjusted to account for timing of vaccination, loss-to-follow-up, and other outcomes. Thus, the denominator at each gestational week precisely calculates the risk only among pregnancies wherein vaccination and no other outcome have occurred. Third, although this analysis was based primarily on participant-report, we reduced misclassification of SABs and perinatal deaths through review of medical records by experienced clinicians among pregnancy outcomes most likely to be misreported.

Our analysis has several limitations. First, some vaccinated women may have participated in the registry specifically to report adverse outcomes (bias away from the null). Others may have opted out of participation to avoid sensitive discussions about adverse outcomes (bias toward the null). Second, the C19VPR cohort may also include participation bias due to healthy vaccinee bias, a phenomenon wherein in general, healthier people participate in vaccination programs [49]. Further, compared to the general US population and previous studies, C19VPR included more women who were younger, White, living in urban areas, and had low-to-normal BMI [22–28]. As a result, it is possible that C19VPR participants are healthier and had differential access to prenatal care when compared with the general population captured through NVSS. Third, our cohort does not include unvaccinated women, resulting in the need to rely on previously published historical data from NVSS for comparison. NVSS has historically documented issues with gestational age reporting, which may result in misclassification [50]. Fourth, the registry cohort is not nationally representative of the US population, limiting generalizability to all vaccinated pregnant women. However, demographic trends in adverse outcomes are similar between the cohort, the general population, and other studies [51–54]. Fifth, while *E*-value analyses suggest our results would not be substantially changed by weak unmeasured confounders, we did not have data for potential sociodemographic confounders including adequacy of prenatal care, insurance status, education, and income level. Inability to account for these factors as well as uncaptured underlying high-risk health conditions may bias effect estimates away from the null and could contribute to the protective associations observed in this analysis. However, an unmeasured confounder associated with both the exposure and outcome by risk ratios of approximately 3.0 would be required to not only eliminate the observed protective effect but indicate harmful association. Further our findings remain consistent with other large studies that have been able to control for confounders missing from our analysis. Sixth, as with all participant-reported data, there is a possibility of measurement error about timing of vaccination, outcomes reported, and gestational age at outcome.

Early data from the COVID-19 pandemic indicated that, similar to other respiratory viral infections [55,56], SARS-CoV-2 infection during pregnancy increases the risk of adverse perinatal outcomes [5]. Limited data on safety of COVID-19 vaccination in pregnancy may have contributed to relatively low COVID-19 vaccination rates among pregnant women [14,15]. As a result, data from post-authorization vaccine safety monitoring systems have been essential in helping patients and providers compare the risks of COVID-19 vaccination and SARS-CoV-2 infection.

## 5. Conclusions

We found no evidence of increased risks of preterm birth or perinatal death among pregnancies exposed to mRNA COVID-19 vaccination prior to or during pregnancy when compared with pre-pandemic estimates of these outcomes. Risks of perinatal death and preterm birth did not differ by vaccine manufacturer. While we could only adjust for age, race, ethnicity, and high BMI, *E*-values suggest that unmeasured confounders would have to be at least moderately associated with both vaccination and perinatal death and strongly associated with both vaccination and preterm birth to substantially change our findings [45].

## Supplementary Material

Supplement

## Figures and Tables

**Table 1:** Participant-reported characteristics among pregnancies ending after 6 weeks’ gestation, CDC COVID-19 Vaccine Pregnancy Registry, January 2021 – September 2022 (*n* = 22,682).

Characteristic	Pregnancies	
	n	%

Age at vaccination (years)		
18–24	373	1.6
25–29	3641	16.1
30–34	10,980	48.4
35–39	6618	29.2
40+	1070	4.7
Race and ethnicity		
Non-Hispanic Black	519	2.3
Non-Hispanic White	17,935	79.1
Hispanic	2148	9.5
Non-Hispanic, Another race	2080	9.2
Parity		
0	10,151	44.8
1	8548	37.7
2+	3983	17.6
Pre-pregnancy BMI (kg/m^2^)		
<18.5	419	1.9
18.5 to 24.9	11,793	52.0
25.0 to 29.9	5952	26.2
≥30.0	4518	19.9
Pre-existing chronic hypertension		
Yes	940	4.1
No	21,742	95.9
Urban resident		
Yes	21,205	93.5
No	1477	6.5
Manufacturer of first COVID-19 vaccine		
Pfizer-BioNTech	13,159	58.0
Moderna	8812	38.9
Janssen	711	3.1
Trimester of registry-eligible vaccine dose		
Pre-pregnancy^a^	2158	9.5
First trimester	6284	27.7
Second trimester	9015	39.8
Third trimester	5225	23.0

a Participants could receive a registry-eligible COVID-19 vaccine dose up to 30 days prior to last menstrual period.

**Table 2:** Week-specific and cumulative risk of perinatal death among participants per 1000 births in the CDC COVID-19 Vaccine Pregnancy Registry (C19VPR) compared to a matched cohort of 2019 National Vital Statistics System data,^a^ January 2021 – September 2022.

Gestational week^b^	C19VPR (n = 22,682)					NVSS 2019 (n = 22,682)				
	Fetuses at Risk^c^	Perinatal deaths	Week-Specific Risk per 1000 births	Cumulative Risk per 1000 births (95% CI)		Fetuses at Risk^c^	Perinatal deaths	Week-Specific Risk per 1000 births	Cumulative Risk per 1000 births (95% CI)	
	*n*	*n*				*n*	*n*			

<18 weeks	10,351	–	–	–	–	22,682	–	–	–	–
18 to <20 weeks	10,950	2	0.2	0.18	(0.00, 0.44)	22,682	5	0.2	0.22	(0.03, 0.41)
20 to <22 weeks	12,184	10	0.8	1.00	(0.44, 1.57)	22,677	23	1.0	1.23	(0.78, 1.69)
22 to <24 weeks	13,391	5	0.4	1.38	(0.72, 2.03)	22,651	27	1.2	2.43	(1.79, 3.07)
24 to <26 weeks	14,676	10	0.7	2.06	(1.28, 2.84)	22,621	16	0.7	3.13	(2.40, 3.86)
26 to <28 weeks	15,836	6	0.4	2.44	(1.60, 3.27)	22,578	14	0.6	3.75	(2.95, 4.54)
28 to <30 weeks	16,867	2	0.1	2.55	(1.70, 3.40)	22,520	12	0.5	4.28	(3.43, 5.13)
30 to <32 weeks	17,822	0	0.0	2.55	(1.70, 3.40)	22,413	5	0.2	4.50	(3.63, 5.37)
32 to <34 weeks	18,750	3	0.2	2.71	(1.84, 3.58)	22,254	11	0.5	4.99	(4.08, 5.91)
34 to <36 weeks	19,485	4	0.2	2.92	(2.03, 3.81)	21,937	11	0.5	5.49	(4.53, 6.46)
36 to <38 weeks	19,636	10	0.5	3.43	(2.49, 4.37)	21,028	13	0.6	6.11	(5.09, 7.13)
38 to <40 weeks	17,246	12	0.7	4.12	(3.10, 5.14)	17,661	16	0.9	7.01	(5.90, 8.12)
40 to <42 weeks	5,919	4	0.7	4.79	(3.57, 6.01)	6,167	7	1.1	8.14	(6.75, 9.52)

a Week-specific and cumulative risk are calculated per 1000 live births and stillbirths at or after that gestational week. Risk is condensed into two-week intervals due to small numbers. Perinatal death was defined as the sum of stillbirths and neonatal deaths within 28 days after birth [38]. Deaths are attributed to the gestational week at which the delivery occurred. Live births resulting in a neonatal death are not mutually exclusive from preterm live births. Pregnancies from 2019 NVSS data from fetal death and birth files were matched to C19VPR based on participant race and ethnicity and age.

b Table begins at earliest reported stillbirth or neonatal death in the registry: 18 weeks’ gestation. C19VPR pregnancies enter as fetuses at risk during gestational week of vaccination. NVSS pregnancies enter as fetuses at risk at immediately.

c Fetuses at risk column represents to number of fetuses included in the denominator for that gestational week. The number at each gestational week is both left censored to exclude fetuses not yet exposed to COVID-19 vaccination and right censored to exclude pregnancies that have already ended (i.e., fetuses at risk = total pregnancies – not yet vaccinated – pregnancy already ended).

**Table 3:** Cox proportional hazard ratios (HR) for risk of perinatal death and preterm birth among CDC COVID-19 Vaccine Pregnancy Registry (C19VPR) participants compared to a matched cohort from 2019 National Vital Statistics System (NVSS),^a^ January 2021 – September 2022.

	Cumulative Risk (95% CI)	Crude HR (95% CI)	Adjusted HR (95% CI)

**Perinatal**			
**Death**^b^			
C19VPR	4.79 (3.57 6.01)	0.58 (0.44, 0.77)	0.62 (0.46, 0.83)
NVSS 2019	8.14 (6.75, 9.52)	[REFERENCE]	[REFERENCE]
**Stillbirth** ^b^			
C19VPR	3.85 (2.76, 4.94)	0.74 (0.53, 1.03)	0.81 (0.57, 1.14)
NVSS 2019	5.04 (3.95, 6.13)	[REFERENCE]	[REFERENCE]
**Neonatal**			
**Death**^c^			
C19VPR	0.95 (0.4, 1.49)	0.31 (0.18, 0.57)	0.33 (0.18, 0.69)
NVSS 2019	3.11 (2.24, 3.98)	[REFERENCE]	[REFERENCE]
**Preterm**			
**Birth**^d^			
C19VPR	6.57 (6.23, 6.92)	0.54 (0.50, 0.58)	0.55 (0.51, 0.58)
NVSS 2019	12.09 (11.67, 12.51)	[REFERENCE]	[REFERENCE]

a Pregnancies from 2019 NVSS data from fetal death and birth files were matched to C19VPR based on participant race and ethnicity and age. Adjusted hazard ratios are adjusted for participant pre-pregnancy body mass index (above and below 30 kg/m^2^), age (continuous), and race and ethnicity (non-Hispanic [NH] Black, NH White, Hispanic, NH American Indian or Alaskan Native, NH Native Hawaiian or Pacific Islander, NH Asian, NH multi-racial, or unknown).

b Risk per 1000 live births or stillbirths after 18 weeks’ gestation, the earliest reported gestational age of delivery among neonatal deaths reported to C19VPR.

c Risk per 1000 live births after 18 weeks’ gestation, the earliest reported gestational age of delivery among neonatal deaths reported to C19VPR.

d Risk per 100 live births after 18 weeks’ gestation, the earliest reported gestational age of delivery among live births reported to C19VPR.

**Table 4:** Week-specific and cumulative risk of preterm birth among participants in the CDC COVID-19 Vaccine Pregnancy Registry compared to a matched cohort from 2019 National Vital Statistics System (NVSS),^a^ January 2021 – September 2022.

Gestational week	C19VPR^b^ (n = 22,203)					NVSS 2019 (n = 22,682)				
	Fetuses at Risk^c^	Preterm Births	Week-Specific Risk	Cumulative Risk		Fetuses at Risk^c^	Preterm Births	Week-Specific Risk	Cumulative Risk	
	*n*	*n*	*%*	*%*	*95% CI*	*n*	*n*	*%*	*%*	*95% CI*

<18 weeks^d^	10,351	–	–	–	–	22,682	–	–	–	–
18 to <19 weeks	10,950	1	0.01	0.02	(0.00, 0.04)	22,682	2	0.01	0.01	(0.0, 0.02)
19 to <20 weeks	11,540	0	0.00	0.02	(0.0, 0.04)	22,680	3	0.01	0.02	(0.00, 0.04)
20 to <21 weeks	12,184	0	0.00	0.02	(0.0, 0.04)	22,677	10	0.04	0.07	(0.03, 0.10)
21 to <22 weeks	12,823	0	0.00	0.02	(0.0, 0.04)	22,667	16	0.07	0.14	(0.09, 0.19)
22 to <23 weeks	13,391	1	0.01	0.03	(0.0, 0.06)	22,651	13	0.06	0.19	(0.14, 0.25)
23 to <24 weeks	13,998	2	0.01	0.04	(0.01, 0.08)	22,638	17	0.08	0.27	(0.20, 0.34)
24 to <25 weeks	14,676	4	0.03	0.07	(0.02, 0.11)	22,621	18	0.08	0.35	(0.27, 0.43)
25 to <26 weeks	15,273	6	0.04	0.11	(0.05, 0.16)	22,603	25	0.11	0.46	(0.37, 0.55)
26 to <27 weeks	15,836	7	0.04	0.15	(0.09, 0.21)	22,578	34	0.15	0.61	(0.51, 0.71)
27 to <28 weeks	16,383	13	0.08	0.23	(0.15, 0.31)	22,544	24	0.11	0.71	(0.61, 0.82)
28 to <29 weeks	16,867	13	0.08	0.31	(0.22, 0.39)	22,520	45	0.20	0.91	(0.79, 1.04)
29 to <30 weeks	17,277	22	0.13	0.43	(0.33, 0.54)	22,475	62	0.28	1.19	(1.05, 1.33)
30 to <31 weeks	17,822	26	0.15	0.57	(0.46, 0.69)	22,413	77	0.34	1.53	(1.37, 1.69)
31 to <32 weeks	18,327	28	0.15	0.73	(0.60, 0.85)	22,336	82	0.37	1.89	(1.71, 2.06)
32 to <33 weeks	18,750	55	0.29	1.01	(0.86, 1.16)	22,254	141	0.63	2.51	(2.31, 2.71)
33 to <34 weeks	19,107	72	0.38	1.38	(1.21, 1.55)	22,113	176	0.80	3.29	(3.05, 3.52)
34 to <35 weeks	19,485	177	0.91	2.28	(2.06, 2.49)	21,937	384	1.75	4.98	(4.70, 5.26)
35 to <36 weeks	19,658	265	1.35	3.59	(3.32, 3.84)	21,553	525	2.44	7.29	(6.96, 7.63)
36 to <37 weeks	19,636	613	3.12	6.57	(6.23, 6.92)	21,028	1,089	5.18	12.09	(11.67, 12.51)

a Pregnancies from 2019 NVSS were matched to C19VPR based on participant race and ethnicity and age. C19VPR pregnancies enter as fetuses at risk during gestational week of vaccination. NVSS data from fetal death and birth files enter as fetuses at risk at immediately.

b C19VPR pregnancies wherein all COVID-19 vaccines were received after 37 weeks’ gestation were excluded from preterm birth analyses (n = 479), resulting in a subsample of 22,203 pregnancies.

c Fetuses at risk column represents to number of fetuses included in the denominator for that gestational week. The number at each gestational week is both left censored to exclude fetuses not yet exposed to COVID-19 vaccination and right censored to exclude pregnancies that have already ended (i.e., fetuses at risk = total pregnancies – not yet vaccinated – pregnancy already ended).

d Table begins at earliest reported live birth in the registry, 18 weeks’ gestation. Live births resulting in a neonatal death are not mutually exclusive from preterm live births; 7 preterm live births that resulted in neonatal death are also included in Table 3 (risk of perinatal death).

## Data Availability

CDC COVID-19 Vaccine Pregnancy Registry data are subject to Assurances of Confidentiality. Data may be made available upon request subject to review.

## Appendix A. Supplementary data

Supplementary data to this article can be found online at https://doi.org/10.1016/j.vaccine.2026.128461.
