# Supplementary material for: Risk of perinatal death and preterm birth among an observational cohort of women vaccinated against SARS-CoV-2 in pregnancy: CDC COVID-19 vaccine pregnancy registry
Source: Vaccine. Author manuscript; Available in PMC 2026 Jun 4. (PMC13234574; doi:10.1016/j.vaccine.2026.128461)
Supplement: Supplement [file NIHMS2175750-supplement-Supplement.docx]

**Supplement 1.** *CDC* *COVID-19 Vaccine Pregnancy Registry survey questions utilized to ascertain spontaneous abortion, perinatal death, and preterm birth.*

| **Question** | **Response options** | |
| --- | --- | --- |
| Our records show you were vaccinated on (dose 1: [dose_1_date] and dose 2: [dose_2_date]). Is this correct?  *Note: dose_1_date and dose_2_date were obtained from CDC V-safe*  If no: Can you please tell me when you received the COVID-19 vaccine? I can give you a minute to look at your vaccination card, if you have it with you.  Corrected dose 1 date:  Corrected dose 2 date: | 1  0 | Yes  No  MM/DD/YYYY  MM/DD/YYYY |
| Our records show you received the (dose 1: [dose1_manufacturer] and dose 2: [dose2_manufacturer]) vaccine. Is this correct?  *Note: dose1_manufacturer and dose2_manufacturer were obtained from CDC V-safe*  If no: Can you please tell me what type of vaccine you received? This should be available on your vaccination card, if you have it nearby.  Corrected dose 1 vaccine manufacturer:  Corrected dose 2 vaccine manufacturer: | 1  0  1  2  3  1  2  3 | Yes  No  Pfizer-BioNTech  Moderna  Janssen/Johnson & Johnson  Pfizer-BioNTech  Moderna  Janssen/Johnson & Johnson |
| What was the start date of your last menstrual period for your pregnancy? |  | MM/DD/YYYY |
| What was your due date? |  | MM/DD/YYYY |
| What was the outcome of your pregnancy? Was it a... | 1  2  3  4  5 | Live birth  Miscarriage  Stillbirth  Induced abortion  Some other outcome (For example, ectopic or tubal or molar) |
| Can you tell me on what date the [outcome] happened?  How many weeks were you at the time of the [outcome]? |  | MM/DD/YYYY  Integer (weeks) |
| Is your child still living?      If no: What was the date of your child’s death? | 1  0 | Yes  No    MM/DD/YYYY |

**Supplement 2.** *Confirmation of Pregnancy Outcomes*

Clinician confirmation of possible stillbirths (spontaneous abortion (SAB) ≥17 weeks’ gestation, stillbirths, and live births with neonatal death within 1 day of delivery; n=95), CDC COVID-19 Vaccine Pregnancy Registry, January 2021 - September 2022

|  | **Total reported** | **Classification after medical record review** | | | | **No records available^a^** |
| --- | --- | --- | --- | --- | --- | --- |
|  |  | **Spontaneous abortion** | **Induced abortion** | **Stillbirth** | **Live birth; neonatal death** |  |
| **Participant-reported pregnancy outcome** |  |  |  |  |  |  |
| SAB ≥17 weeks’ gestation | 28 | 17 | 2 | 0 | 2 | 7 |
| Stillbirth | 61 | 0 | 4 | 43 | 3 | 11 |
| Live birth with neonatal death ≤1 day of delivery^b^ | 6 | 0 | 0 | 0 | 5 | 1 |
| ^a^When no medical records were available, participant-reported pregnancy outcome was retained.  ^b^Neonatal deaths within 1 day of birth were reviewed to confirm whether timing of demise was before or after delivery. | | | | | | |
